# Prenatal anesthetic exposure and offspring neurodevelopmental outcomes—A narrative review

**DOI:** 10.3389/fneur.2023.1146569

**Published:** 2023-03-29

**Authors:** Nannan Zhou, Shuang Liang, Xinying Yue, Wangyuan Zou

**Affiliations:** Department of Anesthesiology, Xiangya Hospital, Central South University, Changsha, China

**Keywords:** anesthesia, pregnancy, maternal exposure, fetus, neurotoxicity, offspring, neurodevelopment, non-obstetric surgery

## Abstract

While it is common for pregnant women to take anesthesia during surgery, the effects of prenatal anesthesia exposure (PAE) on the long-term neurodevelopment of the offspring remain to be clarified. Preclinical animal research has shown that *in utero* anesthetic exposure causes neurotoxicity in newborns, which is mainly characterized by histomorphological changes and altered learning and memory abilities. Regional birth cohort studies that are based on databases are currently the most convenient and popular types of clinical studies. Specialized questionnaires and scales are usually employed in these studies for the screening and diagnosis of neurodevelopmental disorders in the offspring. The time intervals between the intrauterine exposure and the onset of developmental outcomes often vary over several years and accommodate a large number of confounding factors, which have an even greater impact on the neurodevelopment of the offspring than prenatal anesthesia itself. This narrative review summarized the progress in prenatal anesthetic exposure and neurodevelopmental outcomes in the offspring from animal experimental research and clinical studies and provided a brief introduction to assess the neurodevelopment in children and potential confounding factors.

## 1. Introduction

Maternal exposure is defined as the exposure of the female parent (human or animal during pregnancy) to potentially harmful physical, chemical, or biological agents in the environment, such as ionizing radiation, pathogenic organisms, or toxic chemicals, all of which may affect the offspring. The use of an anesthetic is one of the sources of exposure. Prenatal anesthetic exposure (PAE) generally refers to maternal exposure to anesthetic drugs during pregnancy, which occurred in ~0.75–2% of all pregnant women and is one of the types of maternal exposure (1). PAE can be classified clinically under two circumstances. The first case is when the mother herself requires surgery, and in this case, the fetus is similar to an “innocent bystander.” Acute abdominal infections, trauma, and maternal malignancy are the common indications for non-obstetric surgery, while the cesarean section is common for obstetric surgery (1). Advances in assisted reproductive technology have made it possible for more mothers to become pregnant, which indicates that more precious fetuses need to be prevented from being saved from abortion. Therefore, the use of cervical cerclage to prevent abortion also has been on increase in recent years (2, 3). Some of these procedures were performed under general anesthesia. The second case for PAE is where the fetus itself is subjected to a condition that requires an intrauterine surgical procedure. For example, a repair of the spinal meningocele under open feral surgery or fetoscopy requires the mother to undergo general anesthesia.

The main concern about PAE is that the fetus may become vulnerable to maternal anesthetic exposure. According to the standards for grading the harm rendered by drugs to the fetus, classes A and B are safe, while classes C and D show corresponding evidence of the harm rendered to the animal and human fetuses, respectively, but should be used cautiously when the benefits outweigh the harms. The currently available drugs commonly used for prenatal anesthesia are classified as classes B, C, and D. For example, opioids, propofol, and ketamine are widely used clinically, and therefore, more relevant studies are needed to gain an understanding of the neurotoxicity of drugs.

The relationship between PAE and offspring neurodevelopment has attracted more attention of researchers ever since 2016 when the US Food and Drug Administration warned about minimizing the use of narcotics in children under 3 years of age and in mothers during pregnancy (4). A high-quality meta-analysis of animal research showed that anesthesia has neurotoxic effects on the developing brain and impairs learning and memory in the offspring (5). However, due to ethical limitations, evidence from randomized controlled studies (RCTs) is still rare and is largely derived from cohort studies. Therefore, this review summarized the progress in determining the long-term consequences of PAE on the neurodevelopment of offspring, including preclinical and clinical studies.

## 2. PAE and neurodevelopment in offspring of animals

After PAE of the animal models, the neurodevelopment of in the offspring was assessed mainly by observing the morphology of the tissue sections of the central nervous system or by evaluating their cognitive, learning, and memory abilities through some behavioral and maze tests. Rodents and non-human primates are the most commonly used animal models in the field. Prenatal exposure has a significant impact on morphological changes, such as neuronal proliferation, apoptosis, and learning memory capacity, in the offspring of animals, but there are still major limitations of translating the same result to humans.

### 2.1. PAE and neurodevelopment in rodents

Ikonomidou et al. (6) were the first to initiate the treatments of neonatal and pregnant rats with an NMDA antagonist (MK801), and their treatments revealed massive apoptotic degeneration in the brains of the offspring of pregnant rats and of neonatal rats. This study kicked off the debate on the anesthetics and neurotoxicity of animal experiments. It is easier to use volatile anesthetics rather than intravenous anesthetics in mice; therefore, several volatile anesthetics have been used in some animal models. In a study of animals administered isoflurane anesthesia, Rizzi et al. (7) found significant neuronal apoptosis and necrosis in the offspring of guinea pigs exposed to isoflurane for 4 h, with or without nitrous oxide and/or midazolam in early and mid-pregnancy. Palanisamy et al. (8) found that, after administering 1.4% isoflurane anesthesia for 4 h in mid-pregnancy rats, the offspring had impaired spatial memory acquisition. Surprisingly, Nie et al. (9) found that propofol attenuated isoflurane-induced embryonic inflammation, apoptosis, and cognitive impairment in offspring mice.

In a related study about sevoflurane, Zheng et al. (10) used 2.5% sevoflurane to anesthetize mid-pregnancy mice for 2 h and found reduced learning and memory functions in the offspring, along with reduced levels of synaptophysin in the brain tissue, indicating that synaptic integrity was compromised. To investigate whether these impairments were dose-dependent, Wang et al. (11) set different sevoflurane concentrations and anesthetized rats in mid-pregnancy for 2 h. They found that fetal neural stem cell proliferation was inhibited in the high concentration (3.5%) sevoflurane group and that the learning and memory functions were also impaired in postnatal rats. In a study by Zou et al. (12), it was found that, after 3 h of desflurane anesthesia in mid-pregnancy mice, the offspring had impaired memory and lower levels of synaptophysin expression in the brain tissue, suggesting that desflurane exposure during pregnancy may induce memory impairment in the offspring through cell death and disruption of synaptic integrity. Unfortunately, with the introduction of the European Union policies recommending strict restrictions or bans on the use of desflurane to reduce halogenated hydrocarbon emissions causing global warming effects (13), studies on desflurane exposure during pregnancy may be difficult to implement again. Overall, volatile anesthetics have significant neurotoxicity to rodent progeny, while some intravenous anesthetics such as propofol may provide some protection.

### 2.2. PAE and neurodevelopment in non-human primates

Non-human primates are closer to humans in terms of physiology than rodents, and the results obtained are more likely to be translated. Rhesus macaques are the more commonly used animals among non-human primates. Creeley et al. (14, 15) reported that intrauterine exposure to isoflurane or propofol for 5 h in 120-day-old rhesus monkeys resulted in increased apoptosis of neurons and oligodendrocytes. Brambrink et al. (16) set up two types of ketamine exposure in rhesus macaques, respectively, during fetal and early childhood and found that the developing rhesus macaque brain is sensitive to the apoptogenic action of ketamine at both a fetal and a neonatal age. Meanwhile, the loss of neurons attributable to ketamine exposure was 2.2-fold higher in the fetal brain than the neonatal brain, suggesting that prenatal ketamine exposure was more likely to cause neuronal apoptosis. The abovementioned studies on non-human primates also confirmed the toxic effects of PAE on neurons.

### 2.3. Progress in potential confounding factors of animal experiments

The results of animal models could not be directly translated into clinical practice due to the enormous species disparity between humans and other animal models. In addition, animal experiments are often characterized by the following features: first, high doses and long duration of anesthesia; second, only anesthesia without surgery; and third, the absence of vital signs monitoring (e.g., blood pressure). All of these features were different from the anesthesia of humans and therefore would limit the translation to clinical practice. The doses and duration of anesthesia can be changed easily in the experimental environment. Therefore, the surgical procedure itself and intraoperative monitoring during pregnancy become significant potential confounding factors for animal models, and it is crucial to understand how these factors affect the outcomes of the offspring.

The effect of the surgery itself could not be clarified as it was impossible to perform surgical procedures without anesthesia. Bleeser et al. (17) explored the effects of surgery alone by “calculating”. They divided pregnant rabbits into the surgery group under anesthesia and the only anesthesia group. No significant differences were found in neuronal density in the frontal cortex of the offspring between the two groups. This outcome suggested that the abdominal surgery in pregnant rabbits in the second trimester resulted in limited neurophysiological changes but not in neurobehavioral impairments. However, the high stillbirth rate in the surgical group limits translation to the clinical practice because fetal mortality clinically is close to zero. More refined animal models with less severe trauma will be needed in the future.

Hypotension during anesthesia and surgery can be another confounding factor that affects uteroplacental perfusion and therefore the fetus. In this regard, Bleeser et al. (18) used pregnant rabbits to investigate whether hypotension affects the fetal outcome and the neurodevelopment of the offspring. Pregnant rabbits were randomized into a norepinephrine group (maintaining the mean arterial pressure above 80% of baseline) and a hypotensive group and underwent a 2 h exploratory laparotomy under sevoflurane anesthesia. The results suggested no significant difference in frontal neuron density between the two groups of young rabbits. However, fetal survival was significantly lower in the norepinephrine group compared to the hypotensive group, and there were lower sensory scores and less proliferation of caudate and shell nuclei in the neurobehavioral assessment. The results contradict the previous hypothesis that the correction of hypotension is beneficial, possibly due to a reduced blood flow as a result of norepinephrine constricting the uterine vasculature. Future animal models should compare different vital signs (e.g., blood pressure, partial pressure of carbon dioxide, and heart rate) on the offspring outcomes.

## 3. PAE and neurodevelopment of clinical trials

Owing to the ethical restrictions of RCTs, cohort studies are the greatest choice for obtaining high-level information on human maternal anesthesia exposure and neurodevelopmental outcomes in offspring. Data collection on prenatal anesthesia and child neurodevelopmental outcomes have been made possible by database-based regional birth cohorts, which benefit from comprehensive medical records, insurance information, and follow-up methods. This review exclusively discusses prenatal anesthesia for the matrix surgical intervention, excluding PAE for intrauterine fetal surgery.

### 3.1. Regional cohort studies: Early and middle pregnancy

Pregnant women have always been advised to take surgery in the middle of pregnancy due to the risk of induced abortion in early pregnancy. Receiving anesthesia in early pregnancy is mostly due to conditions that should not be delayed, such as maternal emergencies and trauma, or are passively inhaled anesthetic gases while working. An existing study showed that surgery in early pregnancy does not appear to increase the incidence of congenital defects in the fetus, but the abovementioned study did not consider the effect of anesthesia (19). Mid-pregnancy is a period of peak neuronal proliferation and migration and may also be a critical stage affecting neurodevelopment (20). More current clinical studies do not distinguish between early and mid-pregnancy anesthesia; therefore, the PAE presented in this section also mixes anesthesia exposure in early and mid-pregnancy. PAE had an effect on some sub-domains of children's development, but the effect on intelligence was debatable. The relevant study characteristics are shown in Table 1.

**Table 1:** Characteristics of studies on the association between PAE and offspring neurodevelopment.

**Author**	**Year**	**Type of research**	**Type of anesthetics**	**The age of offspring (year)**	**Sample size**	**Primary outcome**	**Secondary outcomes with differences**
Ratzon et al. (21)	2004	Single-center, retrospective matched cohort study	Waste Anesthetic Gases (N_2_O, Halothane, Iso)	5–13	80	Intelligence (-)	Gross motor, ADHD
Creagh et al. (22)	2016	Single-center, retrospective sibling-matched cohort study	N	N	515	ASD (-)	None
Ing et al. (24)	2021	Single-center, retrospective cohort study	General anesthetics	10	2,868	Intelligence (**-**)	Externalizing behaviors
Kravets et al. (23)	2022	Single-center, retrospective cohort study	Fluorinated anesthetics	7	47,977	Intelligence (**+**)	Spelling͙
Bleeser et al. (25)	2023	Single-center, bidirectional matched cohort study	Propofol, Sev et al.	2–18	582	executive function (**-**)	Working memory and attention

PAE, prenatal anesthetic exposure; ASD, autistic spectrum disorder; ADHD, attention deficit and hyperactivity disorder; N, not mentioned in the text; N_2_O, nitrous oxide; Iso, isoflurane; Sev, sevoflurane; externalizing behaviors include impulsivity, aggression, provocation, vandalism, and conduct problems. “+” and “-”, which represent a difference and no difference in the main outcome, respectively. ⁎Note here that the group with PAE instead spells well, which is not consistent with the direction of the intellectual outcome.

Anesthesiologists and nurses working in the operating room may be exposed to anesthetic gases throughout the pregnancy of female patients, which can be viewed as a specific occupational exposure. A cohort study included 40 children aged 5–13 years born to female anesthesiologists and nurses working in the operating rooms (ORs) exposed to waste anesthetic gases, and 40 unexposed children born to female nurses and physicians who worked in hospitals during their pregnancy but did not work in ORs (21). No statistically significant differences were found in the intelligence scores of their children at the age of 5–13 years, but a slightly increased risk of neurological deficits was shown in the exposed group, such as mild motor impairment and inattention/hyperactivity tendencies at school age. This study is the first to answer the question of whether exposure to waste anesthetic gases during pregnancy in anesthesia healthcare workers affects offspring intelligence. However, the anesthetic gases in the study were nitrous oxide, halothane, and isoflurane, which are not the mainstream inhaled anesthetics, and future occupational exposure cohorts need to explore the effect of modern anesthetic gas, such as sevoflurane, on the offspring of anesthesia staff (21).

The sibling-matched cohort led by O'Creagh et al. (22) investigated a total of 515 children, of whom 262 were in the autism spectrum disorder (ASD) group and 99 had been exposed to anesthetics *in utero* or in early childhood; while 253 children of the control group (non-ASD) were siblings of the ASD group and 110 of them had received anesthetics. Exposure rates were similar in both groups, indicating that exposure to anesthetics *in utero* or in early life was not associated with a significantly increased risk of autism and was unlikely to progress to a severe form of the disorder. The study used sibling matching to circumvent the influence of genetics and environment, but the time of exposure included both intrauterine and early life (within 2 years and later), and it was a case-control study in terms of retrospective thinking from outcome to exposure. Kravets et al. (23) explored the relationship between maternal lifetime exposure to surgery or pregnancy exposure to fluorinated anesthetics and children's cognitive development and educational outcomes. The study followed a total of 47,977 children, and some of their mothers had been exposed to fluorinated anesthetics during pregnancy. This study showed that prenatal exposure of children to fluorinated anesthetics was associated with a lower IQ performance in boys at the age of 7; however, these children had better spelling ability. The two correlations mentioned above were in opposite direction, possibly due to the presence of confounding with economic status. Although the sample size of the study was large, only part of the population was PAE. Therefore, one is not likely to conclude that fluorinated anesthetic exposure during pregnancy causes changes in offspring intelligence (23).

The Raine trial included a total of 2,024 mothers and their 2,868 offspring, and the study collected neurodevelopmental indicators of children at 10 years of age (24). The results showed that maternal exposure to general anesthesia during pregnancy was associated with higher scores of externalizing behaviors in the offspring at the age of 10, while no differences were found in other indicators such as intelligence, motor function, and language. The information on exposure for this study was actually obtained from questioning and recalling without strictly taking data from the files and anesthetic sheets. Furthermore, only 22 children were eventually confirmed to have a history of antenatal general anesthetic exposure, far fewer than the 1,994 in the control group, which may have resulted in a greater bias.

The bidirectional cohort study by Bleeser et al. (25) included a total of 129 children with a history of *in utero* anesthetic exposure and 453 controls. They found no significant differences in executive function scores between the two groups, nor for other secondary outcomes such as behavioral problems, psychiatric diagnoses, and learning disabilities. However, differences were found in the two sub-domains of executive function-working memory and attention, when the duration of surgery was >1 h and it was an intra-abdominal surgery. From the multivariate model, the effect of PAE on the executive function and behavioral problem scores were roughly equivalent to the effect of parental educational level, mother's age at birth, and family residence (rented/house). This study, with a more scientific follow-up and estimation of non-response bias, is the largest bidirectional cohort in the field to date. It also had a disadvantage in that, PAE was not the only difference between the two groups. In fact, the vast majority of pregnant women in the anesthetic exposure group received anesthesia for surgery and treatment of diseases (such as tumor surgery or infectious diseases), including possibly CT scans and chemotherapy. Obviously, these treatments have the potential to affect the neurodevelopmental outcomes of the offspring. Overall, for early and middle pregnancy, no significant association was seen between PAE and intelligence, with only minor differences found in motor, behavioral and attentional. It is worth noting that prenatal opioid exposure appeared to be negatively associated with neurocognitive and physical development from the age of 6 months to middle childhood (26, 27).

### 3.2. Regional cohort studies: Late trimester of pregnancy

Anesthetic exposure in late pregnancy mainly includes labor analgesia and cesarean anesthesia, which can be unified as intrapartum anesthesia. Due to the short time between the delivery and anesthesia, it was thought that they would mainly affect neonatal respiration and resuscitation and would not be enough to affect distant neurodevelopment, but there is no evidence to confirm this point. Based on the gaps in the field, some scholars have explored the relationship between intrapartum anesthesia and neurodevelopment in the offspring. Kearns et al. explored the association of general vs. spinal/epidural anesthesia cesarean delivery on developmental outcomes in the first 1,000 days of life (28). They found that cesarean delivery under general anesthesia was associated with neonatal resuscitation, low Apgar, and neonatal hospitalization, but the association with neurodevelopmental test scores at 2 years of age was weak. This study suggested that, in the long term, cesarean delivery under general anesthesia would have little effect on the subsequent development of the offspring compared to neuraxial anesthesia. The study had a sample size of 510,000 and the developmental tests used were non-recognized scales developed within the district, assessing mainly motor and social skills. An earlier cohort study of 5,230 children showed that children born by cesarean section had the same incidence of learning disabilities at the age of 5 years compared to normal birth, suggesting that brief perinatal exposure to anesthetic drugs does not adversely affect long-term neurodevelopmental outcomes (29). Differently, cesarean delivery under neuraxial anesthesia appears to reduce the incidence of learning disabilities compared to normal delivery (no anesthesia) and general anesthesia group, as the incidence of learning disabilities was similar in the normal and general anesthesia groups. The disadvantage was the unevenness between groups due to the small number of cesarean sections (especially cesarean sections under general anesthesia), which may affect the results of the study.

The technology of epidural labor analgesia is quite mature, but there is still a need to increase its popularity. A sibling-matched cohort of over 80,000 children led by Tai Ren et al. (30) showed no association between maternal epidural labor analgesia and specific neurodevelopmental disorders, such as autism and autism spectrum disorders, attention deficit hyperactivity disorder, intellectual disability, and epilepsy, in the offspring. A large sample size and the use of sibling matching in this study provided favorable evidence to support the continued strong promotion of epidural labor analgesia.

### 3.3. Other factors affecting neurodevelopment in offspring

**Figure 1:**
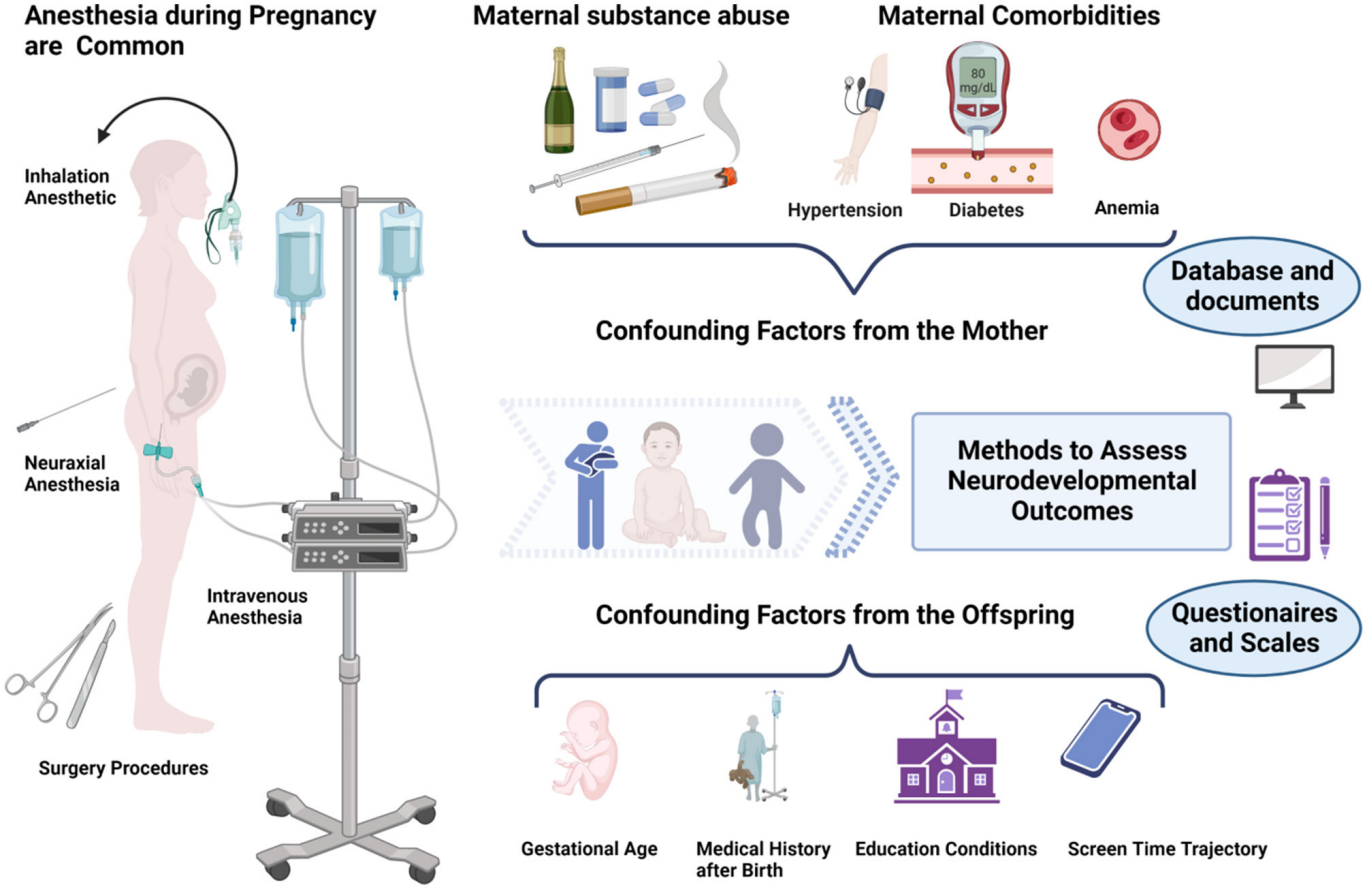
Maternal exposure to anesthesia and surgery during pregnancy may affect the neurodevelopment of the offspring. However, there are many influencing factors to consider ranging from intrauterine exposure to the offspring tests. Figure created with BioRender.com.

The time span between intrauterine exposure and the onset of developmental outcomes varies over several years, which allows confounding factors to come into play. Figure 1 and Table 2 summarize many common potential confounding factors, some of which are well recognized or have been specifically studied in recent years to explore their relationship with neurodevelopmental outcomes in children. For example, some studies found that both eating disorders and anemia during pregnancy were associated with an increased risk of autistic spectrum disorder (ASD) and attention deficit and hyperactivity disorder (ADHD) and that anemia was also associated with intellectual disability (ID) (31, 32). A series of studies shows that cesarean delivery is associated with an increased risk of ASD and ADHD in the offspring (33–35). Other findings include the association between a history of childhood pediatric intensive care unit (PICU) admission and later psychological disorders (36) and the association between early and excessive exposure to electronic devices (such as pads) and lower intelligence scores (37, 38). Information on these variables should be collected in future studies.

**Table 2:** Potential confounding factors of neurodevelopment in offspring.

**Covariates types**	**Specific items**
Maternal comorbidities	Gestational Hypertension, Diabetes mellitus, Hypothyroidism, Epilepsy, Psychiatric disorders, Cardiopulmonary disorders, Anemia (31), Infections, eating disorders (32), etc.
Maternal treatment and therapy	Ionizing radiation, analgesics, antidepressants (39), Anti-tumor drugs
Maternal substances abuse	Alcohol, tobacco, drugs, and their substitutions
Demographic information	Education level and marital status of caretakers, ethnicity, economic level, geographical origin, family conditions
History of birth and disease treatment for offspring	Sex, gestational age, birth weight, delivery by cesarean section (33–35), history of intrauterine distress, history of childhood illness after birth, history of PICU admission (36)
Age and educational background	Children's age at tests, left-behind child, educational condition, screen time on electronics (37, 38)

PICU, pediatric intensive care unit.

It has been reported that the effect values for anesthesia are even smaller than those for other factors such as demographics (25). It is not difficult to understand how a child's good postnatal upbringing may offset a single, small exposure to prenatal anesthesia. Confounding factors are an inevitable problem in such studies. Therefore, collecting relevant indicators in detail and controlling confounding factors will benefit researchers who perform studies of prenatal anesthetic exposure on the neurodevelopment of the offspring.

## 4. Methods of assessing neurodevelopment in offspring

Neurodevelopment in offspring is a vast and complex field involving pediatric and psychiatric mental health science. According to the Diagnostic and Statistical Manual of Mental Disorders-Edition 5 (DSM-5) in the USA, neurodevelopmental disorders (NDDs) mainly include: [1] autistic spectrum disorder (ASD); [2] attention deficit and hyperactivity disorder (ADHD); [3] learning disability (LD); [4] developmental speech or language disorder; [5] developmental co-ordination disorder (DCD); [6] intellectual disability (ID); [7] behavioral disorder; and [8] any NDD (the presence of any of the specific disorders 1–7). As can be observed, intelligence, although a major concern for the general public, is only one branch of neurodevelopmental assessment.

Diagnosing NDD is not easy. When using a medical record system or health insurance database to retrieve diagnostic information, the diagnosis was made in the past by a specialist doctor and scrutinized by the insurance company staff (39). However, when no database is available, the researchers are required to use specialized questionnaires and scales to screen and initially diagnose the study participants in the corresponding areas. For example, in the area of intelligence, the following scales are available: the Wechsler Intelligence Scale for Preschool and Elementary School Children (version of WPPSI) is used to screen preschool children for general intelligence (40); the Raven Colored Progressive Matrices (CPM) are used to assess non-verbal intelligence such as visual and reasoning (24); and the Bailey Scale focuses on assessing intelligence in terms of motor and behavioral responses (41). Similarly, for each sub-domain of NDD, there are relevant assessment methods, such as the CBCL questionnaire and the Conners questionnaire to screen for conduct disorder, the Swanson, Nolan and Pelham-IV rating scales (SNAP-IV questionnaire) to screen for attention deficit hyperactivity disorder (ADHD), and the Autism Behavior Checklist (ABC questionnaire) to screen for autism and ASD. Most of the abovementioned questionnaires and scales are completed by parents. Although being subjective, parental assessments are uniquely valuable in the detection of psychiatric problems in children (42). Depending on the number of questions, questionnaires generally take between a few minutes and an hour to complete and can be completed either online or face-to-face. For some scales that require an observation of children's language, movement, and responses, a face-to-face assessment with scoring by the researcher is necessary. As the most convenient option for researchers, questionnaires and scales are important methods to quantify neurodevelopmental outcomes. Furthermore, understanding the range of applications and choosing scales appropriately can help assess children's neurodevelopmental status as accurately as possible.

## 5. Conclusion

In summary, preclinical animal research has demonstrated that *in utero* anesthetic exposure can lead to morphological changes in the central nervous system, as well as behavioral changes and impaired learning memory capacity in experimental animals. In clinical trials, while no effects of exposure to anesthesia during pregnancy have been found on the intelligence of offspring, minor differences have been found in other areas of neurodevelopment, especially in minor sub-domains such as abnormal behavior and attention. Since much of the clinical evidence comes from cohort studies with numerous confounding factors, future studies should therefore improve the overall cohort quality by enhancing data accuracy and controlling confounding bias.

## Author contributions

NZ contributed to the review of the literature and to the draft of the article. SL and XY revised the manuscript in expressions and grammar. WZ contributed to the draft and played a critical role in manuscript revision. All authors contributed to the manuscript and approved the submitted version.

## References

[1] Reitman E Flood P. Anaesthetic considerations for non-obstetric surgery during pregnancy. Br J Anaesth. (2011) 107(Suppl 1):i72–8. 10.1093/bja/aer343 22156272

[2] Ioscovich A Popov A Gimelfarb Y Gozal Y Orbach-Zinger S Shapiro J. Anesthetic management of prophylactic cervical cerclage: a retrospective multicenter cohort study. Arch Gynecol Obstet. (2015) 291:509–12. 10.1007/s00404-014-3391-5 25103960

[3] Lee A Shatil B Landau R Menon P Smiley R. Intrathecal 2-chloroprocaine 3% vs. hyperbaric bupivacaine 075% for cervical cerclage: a double-blind randomized controlled trial. Anesth Analg. (2022) 134:624–32. 10.1213/ane.0000000000005653 34153006

[4] Andropoulos DB Greene MF. Anesthesia and developing brains—Implications of the fda warning. N Engl J Med. (2017) 376:905–7. 10.1056/NEJMp1700196 28177852

[5] Bleeser T Van Der Veeken L Fieuws S Devroe S Van de Velde M Deprest J. Effects of general anaesthesia during pregnancy on neurocognitive development of the fetus: a systematic review and meta-analysis. Br J Anaesth. (2021) 126:1128–40. 10.1016/j.bja.2021.02.026 33836853

[6] Ikonomidou C Bosch F Miksa M Bittigau P Vöckler J Dikranian K. Blockade of Nmda receptors and apoptotic neurodegeneration in the developing brain. Science. (1999) 283:70–4. 10.1126/science.283.5398.70 9872743

[7] Rizzi S Carter LB Ori C Jevtovic-Todorovic V. Clinical anesthesia causes permanent damage to the fetal guinea pig brain. Brain Pathol. (2008) 18:198–210. 10.1111/j.1750-3639.2007.00116.x 18241241 PMC3886120

[8] Palanisamy A Baxter MG Keel PK Xie Z Crosby G Culley DJ. Rats exposed to isoflurane in utero during early gestation are behaviorally abnormal as adults. Anesthesiology. (2011) 114:521–8. 10.1097/ALN.0b013e318209aa71 21307768 PMC3071297

[9] Nie Y Li S Yan T Ma Y Ni C Wang H. Propofol attenuates isoflurane-induced neurotoxicity and cognitive impairment in fetal and offspring mice. Anesth Analg. (2020) 131:1616–25. 10.1213/ane.0000000000004955 33079886

[10] Zheng H Dong Y Xu Z Crosby G Culley DJ Zhang Y. Sevoflurane anesthesia in pregnant mice induces neurotoxicity in fetal and offspring mice. Anesthesiology. (2013) 118:516–26. 10.1097/ALN.0b013e3182834d5d 23314109 PMC3580035

[11] Wang Y Yin S Xue H Yang Y Zhang N Zhao P. Mid-gestational sevoflurane exposure inhibits fetal neural stem cell proliferation and impairs postnatal learning and memory function in a dose-dependent manner. Dev Biol. (2018) 435:185–97. 10.1016/j.ydbio.2018.01.022 29410165

[12] Zou S Wei ZZ Yue Y Zheng H Jiang MQ Wu A. Desflurane and surgery exposure during pregnancy decrease synaptic integrity and induce functional deficits in juvenile offspring mice. Neurochem Res. (2020) 45:418–27. 10.1007/s11064-019-02932-z 31858378

[13] Hendrickx JFA Nielsen OJ De Hert S De Wolf AM. The science behind banning desflurane: a narrative review. Eur J Anaesthesiol. (2022) 39:818–24. 10.1097/eja.0000000000001739 36036420

[14] Creeley CE Dikranian KT Dissen GA Back SA Olney JW Brambrink AM. Isoflurane-induced apoptosis of neurons and oligodendrocytes in the fetal rhesus macaque brain. Anesthesiology. (2014) 120:626–38. 10.1097/aln.0000000000000037 24158051 PMC3938095

[15] Creeley C Dikranian K Dissen G Martin L Olney J Brambrink A. Propofol-induced apoptosis of neurones and oligodendrocytes in fetal and neonatal rhesus macaque brain. Br J Anaesth. (2013) 110(Suppl 1):i29–38. 10.1093/bja/aet173 23722059 PMC3667347

[16] Brambrink AM Evers AS Avidan MS Farber NB Smith DJ Martin LD. Ketamine-induced neuroapoptosis in the fetal and neonatal rhesus macaque brain. Anesthesiology. (2012) 116:372–84. 10.1097/ALN.0b013e318242b2cd 22222480 PMC3433282

[17] Bleeser T Van Der Veeken L Devroe S Vergote S Emam D van der Merwe J. Effects of maternal abdominal surgery on fetal brain development in the rabbit model. Fetal Diagn Ther. (2021) 48:189–200. 10.1159/000512489 33631746 PMC7613467

[18] Bleeser T Van Der Veeken L Basurto D Valenzuela I Brenders A Van Hoof L. Neurodevelopmetal effects of maternal blood pressure management with noradrenaline during general anaesthesia for nonobstetric surgery in the pregnant rabbit model. Eur J Anaesthesiol. (2022) 39:511–20. 10.1097/eja.0000000000001681 35266919

[19] Colon E Bittner EA Kussman B McCann ME Soriano S Borsook D. Anesthesia, brain changes, and behavior: insights from neural systems biology. Progress in neurobiology. (2017) 153:121–60. 10.1016/j.pneurobio.2017.01.005 28189740

[20] Li X Jiang X Zhao P. Effects of pregnancy anesthesia on fetal nervous system. Front Pharmacol. (2020) 11:523514. 10.3389/fphar.2020.523514 33597861 PMC7883872

[21] Ratzon NZ Ornoy A Pardo A Rachel M Hatch M. Developmental evaluation of children born to mothers occupationally exposed to waste anesthetic gases. Birth Defects Res A Clin Mol Teratol. (2004) 70:476–82. 10.1002/bdra.20044 15259038

[22] Creagh O Torres H Rivera K Morales-Franqui M Altieri-Acevedo G Warner D. Previous exposure to anesthesia and autism spectrum disorder. (Asd): a puerto rican population-based sibling cohort study. Bol Asoc Med P R. (2016) 108:73–80. 29172370

[23] Kravets ME Klebanoff MA Keim SA. Associations between maternal exposure to surgery or pregnancy exposure to fluorinated anesthetics and children's cognitive development and educational outcomes. J Dev Orig Health Dis. (2022) 3:1–10. 10.1017/S2040174422000472 35968856

[24] Ing C Landau R DeStephano D Miles CH von Ungern-Sternberg BS Li G. Prenatal exposure to general anesthesia and childhood behavioral deficit. Anesth Analg. (2021) 133:595–605. 10.1213/ANE.0000000000005389 33497062 PMC9941908

[25] Bleeser T Devroe S Lucas N Debels T Van de Velde M Lemiere J. Neurodevelopmental outcomes after prenatal exposure to anaesthesia for maternal surgery: a propensity-score weighted bidirectional cohort study. Anaesthesia. (2023) 78:159–69. 10.1111/anae.15884 36283123

[26] Yeoh SL Eastwood J Wright IM Morton R Melhuish E Ward M. Cognitive and motor outcomes of children with prenatal opioid exposure: a systematic review and meta-analysis. JAMA network open. (2019) 2:e197025. 10.1001/jamanetworkopen.2019.7025 31298718 PMC6628595

[27] Nelson LF Yocum VK Patel KD Qeadan F Hsi A Weitzen S. Cognitive outcomes of young children after prenatal exposure to medications for opioid use disorder: a systematic review and meta-analysis. JAMA network open. (2020) 3:e201195. 10.1001/jamanetworkopen.2020.1195 32186745 PMC7081119

[28] Kearns RJ Shaw M Gromski PS Iliodromiti S Pell JP Lawlor DA. Neonatal and early childhood outcomes following maternal anesthesia for cesarean section: a population-based cohort study. Reg Anesth Pain Med. (2021) 46:482–9. 10.1136/rapm-2020-102441 33832987

[29] Sprung J Flick RP Wilder RT Katusic SK Pike TL Dingli M. Anesthesia for cesarean delivery and learning disabilities in a population-based birth cohort. Anesthesiology. (2009) 111:302–10. 10.1097/ALN.0b013e3181adf481 19602960 PMC3076711

[30] Ren T Zhang J Yu Y Pedersen LH Wang H Li F. Association of labour epidural analgesia with neurodevelopmental disorders in offspring: a Danish population-based cohort study. Br J Anaesth. (2022) 128:513–21. 10.1016/j.bja.2021.10.042 34893316

[31] Wiegersma AM Dalman C Lee BK Karlsson H Gardner RM. Association of prenatal maternal anemia with neurodevelopmental disorders. JAMA Psychiatry. (2019) 76:1294–304. 10.1001/jamapsychiatry.2019.2309 31532497 PMC6751782

[32] Mantel Ä Örtqvist AK Hirschberg AL Stephansson O. Analysis of neurodevelopmental disorders in offspring of mothers with eating disorders in Sweden. JAMA Netw Open. (2022) 5:e2143947. 10.1001/jamanetworkopen.2021.43947 35040968 PMC8767445

[33] Curran EA Dalman C Kearney PM Kenny LC Cryan JF Dinan TG. Association between obstetric mode of delivery and autism spectrum disorder: a population-based sibling design study. JAMA Psychiatry. (2015) 72:935–42. 10.1001/jamapsychiatry.2015.0846 26107922

[34] Zhang T Sidorchuk A Sevilla-Cermeño L Vilaplana-Pérez A Chang Z Larsson H. Association of cesarean delivery with risk of neurodevelopmental and psychiatric disorders in the offspring: a systematic review and meta-analysis. JAMA Netw Open. (2019) 2:e1910236. 10.1001/jamanetworkopen.2019.10236 31461150 PMC6716295

[35] Zhang T Brander G Mantel Ä Kuja-Halkola R Stephansson O Chang Z. Assessment of cesarean delivery and neurodevelopmental and psychiatric disorders in the children of a population-based swedish birth cohort. JAMA Netw Open. (2021) 4:e210837. 10.1001/jamanetworkopen.2021.0837 33666663 PMC7936261

[36] Ko MSM Poh PF Heng KYC Sultana R Murphy B Ng RWL. Assessment of long-term psychological outcomes after pediatric intensive care unit admission: a systematic review and meta-analysis. JAMA Pediatr. (2022) 176:e215767. 10.1001/jamapediatrics.2021.5767 35040918 PMC8767488

[37] Vohr BR McGowan EC Bann C Das A Higgins R Hintz S. Association of high screen-time use with school-age cognitive, executive function, and behavior outcomes in extremely preterm children. JAMA Pediatr. (2021) 175:1025–34. 10.1001/jamapediatrics.2021.2041 34251406 PMC8276120

[38] Zhao J Yu Z Sun X Wu S Zhang J Zhang D. Association between screen time trajectory and early childhood development in children in China. JAMA Pediatr. (2022) 176:768–75. 10.1001/jamapediatrics.2022.1630 35666518 PMC9171655

[39] Straub L Hernandez-Diaz S Bateman BT Wisner KL Gray KJ Pennell PB. Association of antipsychotic drug exposure in pregnancy with risk of neurodevelopmental disorders: a national birth cohort study. JAMA Intern Med. (2022) 182:522–33. 10.1001/jamainternmed.2022.0375 35343998 PMC8961398

[40] McCann ME de Graaff JC Dorris L Disma N Withington D Bell G. Neurodevelopmental outcome at 5 years of age after general anaesthesia or awake-regional anaesthesia in infancy. (Gas): an international, multicentre, randomised, controlled equivalence trial. Lancet. (2019) 393:664–77. 10.1016/s0140-6736(18)32485-1 30782342 PMC6500739

[41] Reighard C Junaid S Jackson WM Arif A Waddington H Whitehouse AJO. Anesthetic exposure during childhood and neurodevelopmental outcomes: a systematic review and meta-analysis. JAMA network open. (2022) 5:e2217427. 10.1001/jamanetworkopen.2022.17427 35708687 PMC9204549

[42] Rescorla LA Althoff RR Ivanova MY Achenbach TM. Effects of society and culture on parents' ratings of children's mental health problems in 45 societies. Eur Child Adolesc Psychiatry. (2019) 28:1107–15. 10.1007/s00787-018-01268-3 30659384

